# The protective value of the size and movement components of deimatic behavior

**DOI:** 10.1093/beheco/arag047

**Published:** 2026-04-30

**Authors:** Liisa Hämäläinen, Connor M Marsland, Thomas E White, Hannah M Rowland, Kate D L Umbers

**Affiliations:** School of Science, Western Sydney University, Locked Bag 1797, Penrith, NSW 2751, Australia; Department of Biological and Environmental Science, PO Box 35, University of Jyväskylä, 40014 Jyväskylä, Finland; School of Science, Western Sydney University, Locked Bag 1797, Penrith, NSW 2751, Australia; School of Life and Environmental Sciences, The University of Sydney, Camperdown, Sydney, NSW 2006, Australia; Department of Evolution, Ecology and Behaviour, Institute of Infection, Veterinary and Ecological Sciences, University of Liverpool, Biosciences Building, Crown St, Liverpool L69 7ZB, United Kingdom; School of Science, Western Sydney University, Locked Bag 1797, Penrith, NSW 2751, Australia

**Keywords:** defense, deimatism, predator behavior, predator–prey interactions, startle

## Abstract

Deimatic behavior is an antipredator defense such as a sudden movement or sounds performed by prey upon perceiving a threat from a predator. Such behaviors can cause predators to slow or stop their attack, but how different components of deimatic behavior influence predator responses remains untested. We investigated the effect of prey movement, size, and size change on predator attack behavior using a robotic moth and wild Australian magpies (*Gymnorhina tibicen*) as predators. We first tested birds' responses to a nonmoving control moth and then presented them with a moving moth displaying deimatic behavior. The experiment included 3 different deimatic behavior treatments with the moving prey: a small moth, a large moth, and a moth that increased in size from small to large. We found that all deimatic behavior treatments were more effective at stopping the first approach compared with the nonmoving control moth, and that no treatment was more effective than another. There was no significant difference in attack latency among the treatments, although birds tended to attack the prey more quickly after the display when the moving prey remained small, compared with moving prey that increased in size during the movement. The robotic moth did not include warning colors or chemical defenses. Our results therefore indicate that protective value is conferred by movement alone, supporting the “startle-first” hypothesis that the behavioral component of deimatism can evolve before other defenses.

## Teaser

Using a robotic moth and Australian magpies as predators, we show that prey movement alone, without any further defenses, can stop a predator attack when the predator encounters the prey for the first time. This supports the idea that the behavioral component of startle (deimatic) displays can evolve before any other defenses, such as a sudden change from cryptic to warning coloration.

## Introduction

Prey has a diverse range of antipredator defenses (Cott 1940; Ruxton et al. 2018). These defenses include deimatic behaviors that are triggered when the prey perceives a threat from predators (Umbers et al. 2017; Drinkwater et al. 2022). Deimatic behaviors are often multimodal, including visual, auditory, olfactory, gustatory and vibrational modalities, and each modality can also include several components (Rowe and Halpin 2013; Drinkwater et al. 2022). For example, multicomponent visual displays may include a color change (typically a sudden transition from cryptic to conspicuous), a size change (typically an increase in body size), and/or movement (eg Brodie 1977; Martins 1989; Umbers and Mappes 2015; Kang et al. 2017a; O’Hanlon et al. 2018; Chiocchio et al. 2024). Deimatic behaviors were historically suggested to increase prey survival by causing the predator to slow or stop its attack, giving prey time to escape, but this hypothesis has been explored and refined (Drinkwater et al. 2022), and the question of exactly how different aspects of deimatic behaviors protect prey animals is mostly unresolved.

Investigating the protective value of different components of deimatic behavior is crucial for understanding its evolution. Umbers et al. (2017) proposed 2 evolutionary pathways to deimatic behavior. The “defense first” hypothesis suggests that initially camouflaged prey first evolves some form of defense, such as toxicity, which then facilitates the evolution of further defenses, such as conspicuous color signals (Umbers et al. 2017). In the defense-first hypothesis the detection cost of conspicuousness is offset by hiding the signal and revealing it only when the prey perceives a threat from predators, leading to deimatic behavior. Alternatively, the “startle first” (or “behavior first”; Drinkwater et al. 2022) hypothesis suggests that behavior, such as movement of wings during the initial phase of escape from predators, can alone increase prey survival, and this effect can be enhanced by further defenses, such as chemical defenses and conspicuous color signals (Umbers et al. 2017). To test the feasibility of these 2 evolutionary pathways, we need to isolate the effects of different components of deimatic behavior on predator deterrence.

Predator responses to some components of deimatic behavior have been experimentally tested. For example, many insects produce sounds in response to a threat (Low et al. 2021), and predation experiments have shown that the sound alone can slow or stop the attacks from avian (Dookie et al. 2017) and mammalian predators (Olofsson et al. 2012). Another component that has been well studied is color change from cryptic to conspicuous, which increases the protective value of deimatic behaviors in at least some contexts (Kang et al. 2017b; Holmes et al. 2018). Studies involving color change often also include other visual components, such as movement and an increase in size (Kang et al. 2017b; Holmes et al. 2018) which could have interactive effects. The protective effects of movement and size change, however, remain largely unexplored, and their individual and combined impacts on predators are unknown.

Body size alone can be an important component in the protective value of prey defenses including deimatic behavior (Skelhorn et al. 2016). Kang et al. (2017b) found that both startle responses of avian predators and the survival of prey were higher in larger than in smaller artificial prey performing deimatic behavior. Furthermore, hidden coloration is associated with larger body size in several insect taxa (Kang et al. 2017b; Loeffler-Henry et al. 2019; but see Vidal-García et al. 2020), possibly because larger species are easier to detect and might be under stronger selection pressure for additional defences (Kang et al. 2017b). Deimatic behavior might also be more efficient in larger species because such species look more intimidating (Kang et al. 2017b). The effect of apparent size change resulting from performing the behavior, however, has not been tested even though many deimatic behaviors include a body size increase (eg by opening wings or inflating the body, Drinkwater et al. 2022). Similarly, the effect of movement is understudied, although there is some evidence that continuous movement, also called “rhythmical deimatic behavior” (Blest 1958), can deter predators without the need for any further defenses (Holmes et al. 2018). Holmes et al. (2018) found that domestic chicks (*Gallus gallus domesticus*) hesitated longer to attack video prey when the forewings of the prey were repeatedly opening and closing at fast speed, but this effect was not significant when prey moved at slow or moderate speed. Furthermore, the study tested for the effect of continuous wing flicking, and while this is observed in some species (eg in a peacock butterfly *Inachis io*, Vallin et al. 2005), many deimatic behaviors include only one sudden movement, such as when the prey opens its wings and holds that position (e.g. *Catocala* moths, Schlenoff 1985; a mountain katydid *Acripeza reticulata*, Umbers and Mappes 2015; a spotted lanternfly, *Lycorma delicatula*, Kang et al. 2017a). Whether this type of noncontinuous movement can provide protection without further defenses therefore remains untested.

Most experimental studies on deimatic behavior have tested the responses of predators during their first encounter with the behavior (Drinkwater et al. 2022), but to understand the protective value of different components it is also important to investigate how predator responses change across repeated encounters with prey (Sherratt 2011; Sherratt et al. 2023). Change in response is likely to depend on several factors, such as whether prey possess some form of chemical defense, and how often predators encounter the prey (Drinkwater et al. 2022). For example, Kang et al. (2016) showed that oriental tits (*Parus minor*) learned to avoid chemically defended prey that revealed conspicuous color patterns during deimatic behavior, and the learning speed was similar to that after the exposure to aposematic prey with constant conspicuous coloration. Conversely, laboratory studies with wild-caught blue jays (*Cyanocitta cristata*) and artificial moths have shown that birds can habituate to (learn to ignore) deimatic behavior that does not include any chemical defenses (Schlenoff 1985; Ingalls 1993). These studies indicate that the habituation process depends on the number of different color signals in the population of prey that performs deimatic behavior, with a higher signal diversity increasing the habituation time, and that there are consistent individual differences among bird predators in how quickly they habituate (Ingalls 1993). However, habituation to deimatic behavior in laboratory experiments with captive predators and artificial prey may be different to habituation in the wild, where there is more alternative prey and where the encounter rates with prey might be lower. Studies investigating predator habituation in the wild are scarce. Umbers et al. (2019) found that previous experience influenced how wild Australian magpies (*Gymnorhina tibicen*) responded to the deimatic behavior of mountain katydids, with sympatric predators regularly consuming chemically defended katydids and naïve allopatric predators avoiding them. While this study provided evidence that predators can learn to ignore deimatic behavior in the wild, determining how quickly this happens and which components of the deimatic behavior are important to prevent predator habituation requires further investigation. Finally, the protective value of movement and apparent size change in repeated encounters in the absence of color signals has not been tested.

Here, we conducted a field experiment with Australian magpies to investigate both predator initial responses and habituation to 2 components of deimatic behavior. We tested how prey movement, size, and increase in size influence predator behavior using an artificial prey, a robotic moth with opening forewings (inspired by Kang et al. 2017b). Because it is already well established that color pattern signals enhance the effect of deimatic behavior (Kang et al. 2017b; Holmes et al. 2018; Riley et al. 2023) and because our study focused on movement, size, and size change, the moth was gray and did not include any typical warning colors. Each bird was presented with a nonmoving control moth and then received one of the 3 deimatic behavior treatments that included different combinations of movement, size, and size change: (i) a small moth with movement but no change in size (small), (ii) a large moth with movement but no change in size (large), and (iii) a moth that increased in size from small to large with movement (small-to-large). The combination of the nonmoving control moth and the 3 treatments allowed us to disentangle the effect of movement with and without size change. Isolating the effect of size change alone without movement is not possible, as the increase in size always includes some movement. Decrease in size was not included in the experiment because this is not typically observed in deimatic behaviors (Drinkwater et al. 2022). In the experiment, magpies were presented with the same deimatic behavior treatment until they either habituated to the movement (ie attacked the prey and showed no aversive behavioral responses), or until they refused to approach the prey again. We predicted that size change would increase the protective value of deimatic behaviors by increasing prey avoidance during the first encounter and by more often preventing predator habituation, leading to refusal to revisit the otherwise rewarding prey. Our experiment also allowed us to investigate if movement alone, without size change, any warning colors, or chemical defenses, would deter predators. If so, this would provide evidence for the startle first (or behavior-first) hypothesis of the evolution of deimatic behavior.

## Methods

### Predators

We used wild Australian magpies as predators in this experiment (*n* = 32). The Australian magpie (Indigenous names: Djarrawunang, Wilbung, Marriyang) is a large passerine bird that is common in urban and suburban areas and is easily habituated to humans (Kaplan 2004). Magpies are generalist feeders that forage on the ground, and they are therefore potential predators for insects displaying deimatic behavior. They live in small family groups in stable territories (∼2 km^2^) that rarely overlap with those of other groups (Kaplan 2004). We conducted the experiment in greater Sydney, Australia in public parks and university campuses. Because our aim was to conduct repeated trials with the same bird, it was essential that we could identify individual magpies. We therefore captured some of the individuals (*n* = 15) before the experiment using a walk-in trap baited with mozzarella cheese. The captured birds were fitted with individually numbered ABBBS metal bands and plastic colored bands that allowed individual identification. Often magpies could be identified without banding based on their high territoriality and individual plumage markings, and we also included unbanded magpies (*n* = 17) in the experiment when we could confidently identify them. After the experiment, all magpies were weighed by encouraging them to hop on a scale (Ohaus Valor 7000) by placing pieces of cheese on the tray that they could not resist.

### Robotic prey

We investigated predator responses to deimatic behavior using a robotic moth with moving forewings. The wings were made of paper, and they were attached to 2 rotating servos (Arduino Compatible 9G Micro Servo Motor) with Blu Tack (Bostik). The movement was controlled with a push button that was connected to an Arduino board (Arduino UNO R3) with >15 m copper wire, which allowed the experimenter to observe the magpie from a distance without disturbing it. In each case, the angle between the initial position of the moth's wings and the position after the movement was 60°, and the time to complete this movement was ∼0.05 s. The robotic moth was presented on top of a wooden box (179 × 120 × 77 mm), and all the electronics were inside the box. Because the mechanical noise of the rotating servos could not be eliminated, the birds were habituated to the noise before the experiment to ensure that it would not influence their responses (see Experimental protocol).

**Figure 1:**
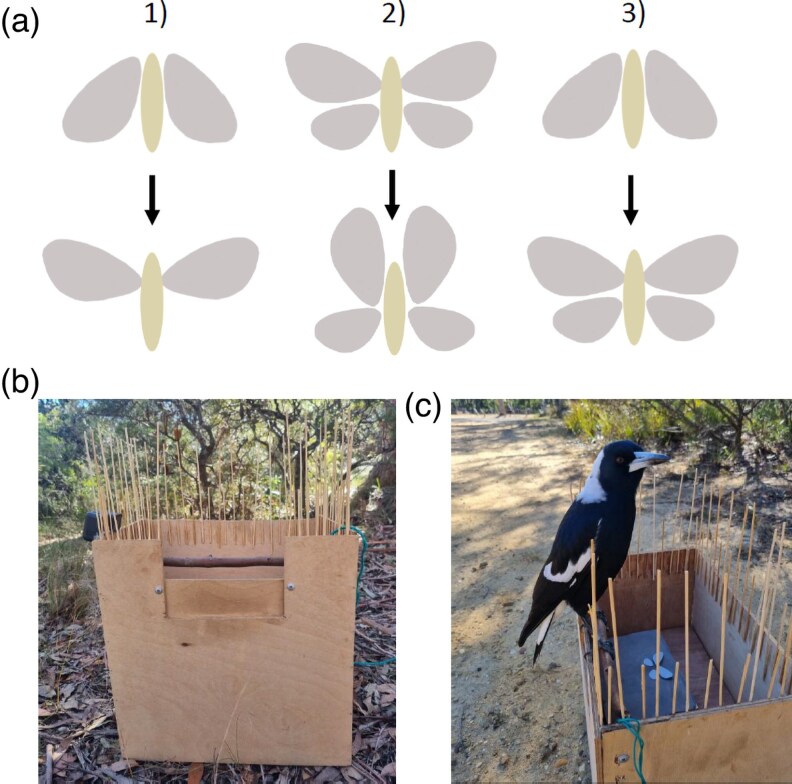
Experimental set-up showing a) the 3 deimatic behavior treatments in the experiment: (1) a small moth with movement but no change in size (small), (2) a large moth with movement but no change in size (large), and (3) a moth that increased in size from small to large with movement (small-to-large); b) the experimental arena with dark brown stick perch; and c) a magpie standing on the perch while participating in the experiment.

Moth fore- and hindwings were cut from light gray paper (reflectance 36.09%), so that they resembled the shape of *Catocala* moth wings. The surface area of each forewing was 350 mm^2^ (30 mm from the base to the tip of the wing) and of each hindwing 183 mm^2^ (20 mm from the base to the tip), which fell in the size range of butterflies and moths observed in the study area (Zborowski and Edwards 2007). The total wing surface area of the moth was 1,066 mm^2^ when both fore- and hindwings were visible, and 700 mm^2^ when only forewings were visible (in the small treatment and when forewings were closed, Fig. 1a). The background was a dark gray paper (reflectance 12.93%) that was glued on top of the wooden box where the moth was presented. The difference in reflectance between the moth and the background (∼23%) made the moth detectable to birds even though it did not feature any typical warning colors. Hindwings were attached to the background with Blue Tack (in large and small-to-large treatments, Fig. 1a), and the forewings were attached to the rotating servos. In the experiment, a piece of mozzarella cheese (∼25 mm long) was placed between the wings to simulate a moth body and encourage birds to approach and attack.

### Experimental arena

The robotic moth was presented to birds in a 37 × 25 × 37 cm sized experimental arena that was made of plywood (Fig. 1b). The small box with the robotic moth was placed on a plywood tray inside this arena, so that it could not be seen from the ground. Along the inside of one side of the arena we attached a stick (∼2 cm in diameter) to serve as a perch. To see and attack the moth, birds had to hop onto this perch and lean towards the moth. Around the remaining periphery of the arena, we attached in a vertical position and at ∼2 cm intervals wooden skewers 16 and 7 cm in length, which prevented birds from landing on the arena anywhere other than the perch (Fig. 1b). This was necessary to ensure that birds always approached the prey from the same direction, and to facilitate recording of the moment when birds first saw the moth. The design of the arena also minimized opportunities for social information use as only the bird on the perch could see the moth. However, other birds could still observe the behavioral responses of the bird that participated in the experiment. During the experiment, the arena was placed in the shade to make the lighting as standardized as possible across the arena and the moth. To prevent birds from seeing the prey from above, we also avoided placing the arena directly under the trees where the birds were perching.

### Magpie pretraining

Before the experiment, birds were trained to associate the arena with a food reward and to hop onto the perch. This was achieved by first offering mozzarella cheese in front of the experimental arena, then inside the experimental arena so that the food reward was visible from the ground (by lifting up the tray inside the arena to make it visible), and finally so that the food reward could be seen only when birds were on the perch (similar to the experimental trials). During the training, the cheese was offered on top of the same wooden box that housed the robot for the experiment but without the gray background paper. Training continued until the bird hopped onto the perch twice in a row and ate the cheese. If several birds from the same family group approached the arena, they could be trained at the same time, but each bird had to individually complete the training (hop onto the perch twice) before testing. Because some birds completed the training more slowly than others (or did not complete it), it was possible that some birds in the group were tested while others were still training. In this case, we used cheese to lure other birds away from the arena, so that they would not affect the experimental trial or acquire social information.

### Experimental protocol

The experimental trials were conducted from May to July 2023. Each bird was randomly allocated to one of the 3 deimatic behavior treatments with moving prey: (i) small (*n* = 10), (ii) large (*n* = 11), and (iii) small-to-large (*n* = 11, Fig. 1a, see Supplementary Videos). In small and small-to-large treatments the robotic moth was first presented to birds with forewings closed, and the display consisted of the forewings opening (moving upward 60°). In the small-to-large treatment, this movement revealed hidden hindwings, whereas in the small treatment, the moth did not have hindwings, so the display included only movement and no increase in size. In the large treatment, the moth was first presented with forewings open, so that both forewings and hindwings were visible, and the display consisted of the forewings moving upward 60°. This movement was similar to the other 2 treatments, but both fore- and hindwings remained visible throughout the display. The size of the moth in the initial position (before movement) was therefore larger in this treatment compared with small and small-to-large treatments, but there was no size change during the movement.

Before birds received a deimatic behavior treatment with moving prey, they were all presented a nonmoving control moth. The control moths were presented in the initial positions of each treatment (top row of Fig. 1a), with forewings closed in the small and small-to-large treatments, and open in the large treatment. This ensured that a nonmoving control moth matched the “before movement” position of the moth for the 3 treatments, and the addition of the movement was the only difference between the control and the experimental trials. Because the mechanical sound of the robot might influence bird responses, the control treatment included the same sound. This was achieved by placing the rotating servos inside the wooden box, so that birds could hear the rotating sound but did not see the moving parts of the robot. This ensured that the birds became habituated to the mechanical sound before the trials with the moving prey, so that any differences in bird behavior between moving and nonmoving moths could not be explained by the sound.

To encourage magpies to approach the arena during the experiment, 1 piece of mozzarella cheese was placed on the ground directly in front of the perch. The experiment was started after other birds than the target individual (other magpies or other species such as cockatoos or butcher birds) were lured away from the arena with cheese, so that they would not interfere with testing. The target individual typically ate the cheese in front of the arena first and then hopped onto the perch. To standardize the timing of the robot movement (or sound in the control trials), the robot wings were opened the moment the bird landed on the perch. This was controlled by an experimenter (using a push button) who observed the bird from 2 to 5 m distance. Most of the birds hopped directly onto the perch; however, in some cases the birds first tried to approach the prey from the other sides of the arena but could not land there because of the wooden skewers on the edges (Fig. 1b). In these cases, we waited for the birds to hop onto the perch (Fig. 1c) before presenting them with the display so that all birds perceived the prey behavior from the same angle.

Before the experimental trials with the moving prey, all birds had to complete 2 consecutive control trials where they attacked the nonmoving prey (ie ate the cheese) without any observed behavioral responses to the mechanical sound. These behavioral responses included hopping off the perch onto the ground without eating the cheese, hopping up and down on the perch, leaning backwards on the perch and flapping wings, or giving an alarm call. If the bird performed any of these behaviors, the control prey was presented again until birds became habituated to the sound and no longer responded to it. In each trial, we presented the sound only once when birds landed on the perch for the first time, and the trial was completed when the bird ate the cheese. If the bird left the perch without eating the cheese, we waited for 5 min for it to return. If the bird returned, it was allowed to eat the cheese without the sound being presented again to ensure that the bird would get a positive reward and be more likely to return to the arena. If the bird did not return in 5 min, the experiment was paused for at least 10 min before the control prey was presented again for 15 min at a time, until the bird approached and attacked the prey. After the bird had completed the first control trial (ie eaten the cheese), we presented the control prey again with the mechanical sound, and this was continued until the bird attacked the prey in 2 consecutive trials without showing any responses to the sound. Four birds did not return to the arena after the first encounter with the control moth, and they were not used in the experiment.

After the bird had completed 2 control trials without behavioral responses, we started the experimental trials where the birds were presented with the deimatic behavior treatments. We followed the protocol from the control trials, presenting the display only once in each trial, and recorded whether birds remained on the perch and attacked the prey (ate the cheese) immediately after the display. If the bird hopped out of the arena without attacking the prey, the wings were left in the display position (forewings moved upward) and the bird was given 5 min to approach the prey again. The movement was not performed again until the bird had returned and finished the trial (eaten the cheese). We then closed the wings and proceeded to the next trial, in which the same moving display was presented again. This was continued until the bird either (i) attacked the prey in 2 consecutive trials without any observed behavioral responses to the movement (habituation), or (ii) refused to hop back onto the perch in 4 consecutive 15 min long trials (rejection). In the second option, the bird was required to eat the piece of cheese that was placed in front of the arena to show that it was still food-motivated and aware of the availability of food, and 15 min was counted from that time point. If the bird was in the area but did not approach the arena and eat the cheese in front of it, this was not counted as a trial. This meant that if the bird did not hop onto the perch, we could conclude that it was because the bird was hesitant to approach the prey again, rather than not being motivated to forage. Because we conducted the experiment in the wild, standardizing the number of trials conducted on each day was not possible, as this number depended on birds' motivation to forage and weather conditions. Most of the birds (*n* = 22) completed both control and experimental trials within 3 consecutive days, but in some cases (*n* = 10), this took 4–9 d. One bird took 17 d to finish the experiment because it was not observed for several days and at times was not motivated to approach the arena.

All trials were filmed with an HD camcorder (Sony HXR-NX30P) placed ∼1 m from the arena, and with a GoPro (Hero 4 Silver) attached to the edge of the arena. We quantified from the videos any behavioral responses to the display (see above). In addition, we measured how long it took for birds to attack the prey. If the bird did not attack immediately after the display or approach the prey again during the same trial, we waited for the bird to eat the cheese placed in front of the arena in the following trial and continued to measure the latency to attack the prey from that time point. This confirmed that the birds were in close proximity to the arena and motivated to forage, so any time delays to approach the prey were due to birds' hesitation to approach it again, rather than their lack of motivation.

### Statistical analyses

#### Control vs. experimental trials

To investigate the effect of movement on prey survival, we compared birds' responses during their first encounter with the nonmoving control prey and during the first presentation of the moving prey in the experimental trials. We used a generalized linear mixed effect model with a binomial error distribution and logit link to analyze whether birds were more likely to attack the control prey compared with the experimental prey (*n* = 32). The response variable was coded 1/0 depending on if the bird attacked the prey immediately after the first encounter or not. Trial type (control/experimental) was included as a fixed effect and bird identity was included as a random effect to control for repeatable measures from the same individuals. In addition, we compared birds' latencies to attack the control and experimental prey using a mixed-effects Cox proportional hazard model (*n* = 32). Time (s) to attack the prey after the first encounter was used as a response variable, trial type (control/experimental) was included as a fixed effect and bird identity as a random effect. If the bird did not attack the prey immediately, time to attack was recorded as the time to return to the prey after the first encounter (during the same trial or the following trials). The birds that hopped away after the first moving display and did not approach the prey again during the experiment (*n* = 5) were right censored with a value of 3,900 s which was the maximum time given for birds to return to the prey, comprising 5 min after the first display followed by 4 × 15 min trials.

#### First trial with the moving prey

We next investigated birds' responses to different deimatic behavior treatments during the experimental trials. We used generalized linear models with a binomial error distribution and logit link to analyze (i) whether birds attacked the prey immediately after the first encounter (*n* = 32), and (ii) if they approached the prey again within 5 min from the first encounter (if not attacking immediately, *n* = 22). In both models we specified the response variable as binomial (coded 1/0 depending on if the bird attacked/approached again or not) and included deimatic behavior treatment (small/large/small-to-large) and birds' mass (g) as fixed effects. Birds' mass was included in the models to represent birds' body condition, which could influence their motivation to forage and approach the prey. Because there were no differences among the 3 treatments (see Results), we also ran additional models to further investigate the effect of size change. These models were otherwise similar to the ones described above but instead of treatment, the fixed effect was size change with 2 levels: no size change (combining small and large treatments where prey did not increase in size) and increase in size (small-to-large treatment).

#### Predator behavior after the first encounter with the moving prey

To analyse whether birds returned to the prey after the first encounter with the moving display (during the same or the following 4 trials), we used a generalized linear model with a binomial error distribution. The response variable was coded 1/0 (returned or did not return), and deimatic behavior treatment and birds' mass were included as fixed effects. This analysis included all birds, regardless of whether they attacked the prey already during the first encounter (*n* = 32). Again, we also ran a separate model with size change (no size change/increase in size) as a fixed effect, instead of treatment. In addition, we analysed the latency to attack the prey after the first encounter using a Cox proportional hazards model. Time (s) to attack the prey after the first moving display was used as a response variable, and deimatic behavior treatment was included as the explanatory variable. The model included all individuals (*n* = 32): the birds that attacked the prey immediately after the first moving display (time to attack after the display was presented), as well as those that hopped away after the first display (time to return to the prey after the display). The birds that hopped away and did not approach the prey again during the whole experiment (*n* = 5) were again right censored with a value of 3,900 s.

#### Predator habituation

We used a generalized linear model with a binomial error distribution to analyse whether birds habituated to the moving display, that is, attacked the prey in 2 consecutive trials without any measurable behavioral responses (coded 1/0 depending on whether birds habituated or not, *n* = 32). We again ran 2 separate models, with deimatic behavior treatment and birds' mass, or size change and birds' mass as fixed effects. We also counted how many times birds approached the moving prey before either habituating or rejecting it. This included the first encounter with the moving prey and the number of times the bird approached the prey again before reaching the criteria for habituation or rejection. Because the sample sizes for both measures were low (habituation: *n* = 14, rejection: *n* = 18), we analyzed differences among treatments using a Kruskal–Wallis test. Finally, we used a Spearman's correlation test to investigate if the number of encounters with the moving prey before habituation was associated with the habituation speed to the robot sound during training (the number of training trials where the bird showed a behavioral response to the nonmoving prey with sound, *n* = 14).

All analyses were conducted in R version 4.4.3 (R Core Team 2025). The GLMs were conducted using the *lme4* package (Bates et al. 2015) and the significance of the main effects in the models was tested using the Anova() function in the *car* package (Fox and Weisberg 2019). Cox proportional hazards models were conducted using *coxme* (Therneau 2024a) and *survival* packages (Therneau 2024b). The graphs were made using the *ggplot2* (Wickham 2016) and *survminer* (Kassambara et al. 2025) packages.

## Results

### Control vs. experimental trials

**Figure 2:**
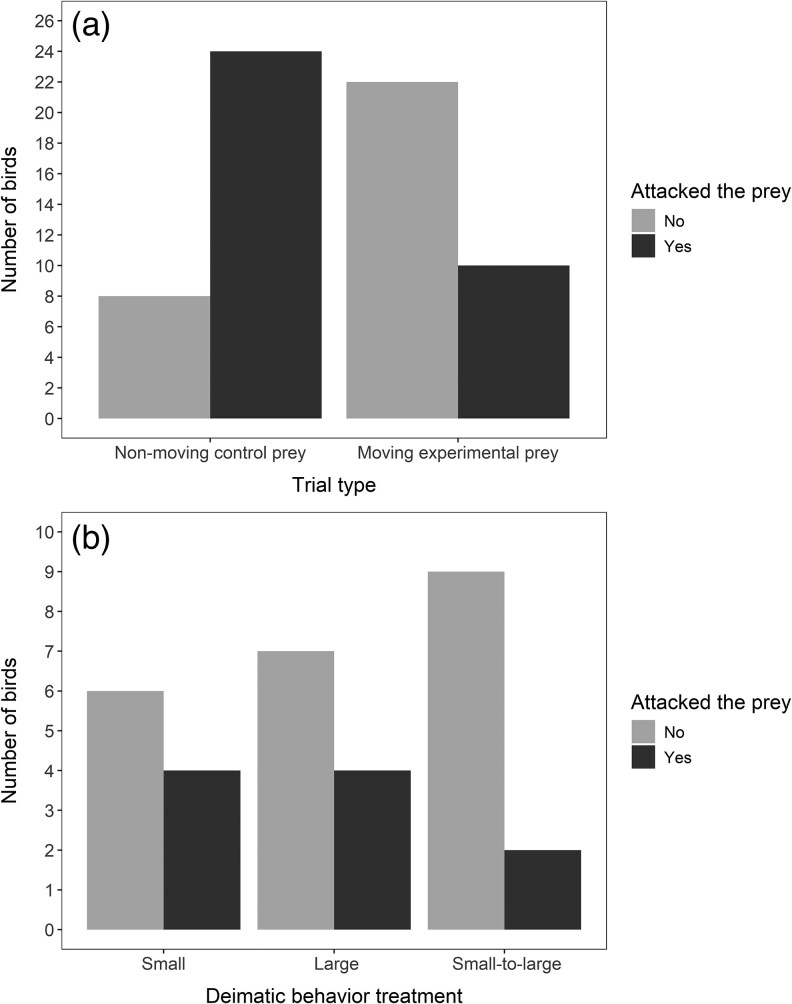
Birds’ attack decisions when encountering the prey for the first time. a) Birds’ (*n* = 32) responses to the first presentation of the nonmoving control prey (with the robot sound) and to the first presentation of the same prey in motion. b) Birds’ first responses to the different deimatic behavior treatments of the moving prey (small: *n* = 10, large: *n* = 11, small-to-large: *n* = 11).

Birds were significantly more likely to attack the prey during the first control trial with the nonmoving moth (75% attacking), compared with the first experimental trial with the moving moth (31% attacking, GLMM: the effect of movement = −20.345 ± 4.060, *Z* = −5.011, *P* < 0.001, Fig. 2a). Similarly, birds attacked the control prey more quickly than the moving experimental prey (coxme: the effect of movement = −1.278 ± 0.315, *Z* = −4.06, *P* < 0.001). Most of the birds did not show any behavioral responses to the robot sound during the first 2 control trials (*n* = 15) or only performed a small wing flap (*n* = 7) but remained on the perch to eat the cheese (Fig. 2a). However, 8 birds hopped onto the ground without eating the cheese when presented with the control prey, and the control trials were continued until the birds became habituated to the robot sound, which took 3–10 trials (mean = 6).

### First trial with the moving prey

When birds encountered the moving experimental prey for the first time, most hopped away from the perch onto the ground without attacking the prey (Fig. 2a). The proportion of birds that attacked the prey immediately after the movement did not differ among the 3 deimatic behavior treatments (GLM: χ^2^ = 1.643, df = 2, *P* = 0.44, Fig. 2b), and the attack decision was not influenced by the bird's mass (GLM: χ^2^ = 0.206, df = 1, *P* = 0.65). We also found no evidence of the apparent size change influencing birds' attack decisions when analysing it separately (GLM: the effect of size change = −1.157 ± 0.966, *Z* = −1.198, *P* = 0.23). Most of the 10 birds that attacked the prey immediately still showed some behavioral responses to the moving display, including flapping their wings (8 birds), performing a small hop on the perch (1 bird) and giving an alarm call (1 bird). If birds did not attack the prey immediately after the movement (*n* = 22), they were given 5 min to return to eat the food reward. The decision for the birds to approach the prey again within 5 min did not differ among the treatments (GLM: χ^2^ = 1.729, df = 2, *P* = 0.42) and was not influenced by the bird's mass (GLM: χ^2^ = 0.335, df = 1, *P* = 0.56). Similarly, this was not explained by the apparent size change of the prey (GLM: the effect of size change = −1.302 ± 1.062, *Z* = −1.226, *P* = 0.22).

### Predator behavior after the first encounter with the moving prey

**Figure 3:**
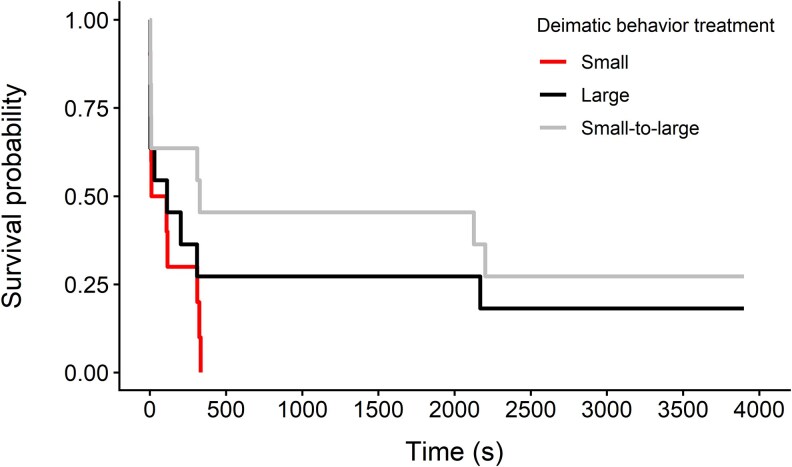
Prey survival (i.e. time before birds attacked the prey) after the first moving display (small: *n* = 10, large: *n* = 11, small-to-large: *n* = 11). Birds that did not approach the prey again after the first encounter (*n* = 5) were right censored.

Most of the birds (26 of 32) approached the prey again after the first encounter with the moving display, and this decision to return did not differ among the deimatic behavior treatments (GLM: χ^2^ = 1.701, df = 2, *P* = 0.43) or depend on the bird's mass (GLM: χ^2^ = 0.936, df = 1, *P* = 0.33). Similarly, the apparent size change of the prey did not explain whether birds approached the prey again (GLM: the effect of size change = −1.155 ± 1.022, *Z* = −1.129, *P* = 0.26). There was a tendency, although not significant, for birds to attack the prey more quickly when the moving prey was small, compared with the prey that increased in size (coxph: the effect of small-to-large treatment = −0.969 ± 0.497, *Z* = −1.949, *P* = 0.051, Fig. 3). There was no significant difference in the latency to attack the prey between the small and large treatments (the effect of large treatment = −0.499 ± 0.474, *Z* = −1.053, *P* = 0.29), or between the large and small-to-large treatments (the effect of small-to-large treatment = −0.470 ± 0.489, *Z* = −0.962, *P* = 0.34, Fig. 3).

### Predator habituation

**Figure 4:**
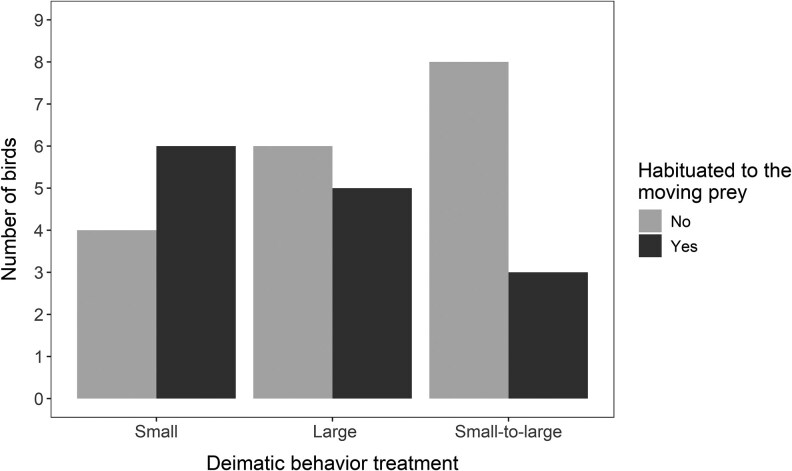
Birds’ habituation to the moving prey during the experiment (small: *n* = 10, large: *n* = 11, small to large: *n* = 11).

There was no significant difference among the 3 deimatic behavior treatments in habituation to the prey movement (GLM: χ^2^ = 4.885, df = 2, *P* = 0.087). When the apparent size change was analysed separately, there was a tendency for birds to be less likely to habituate to the movement when the prey increased in size but this effect was not statistically significant (GLM: the effect of size change = −1.840 ± 0.999, *Z* = −1.842, *P* = 0.066, Fig. 4). We also found a nonsignificant trend for heavier birds to be more likely to habituate to the movement (GLM: χ^2^ = 3.825, df = 1, *P* = 0.050). In addition to the 14 birds that reached our criterion of habituation, there were 4 birds (small: *n* = 1, large: *n* = 1, small-to-large: *n* = 2) that attacked prey in one of the movement trials, but hopped away after the movement in the following trials, or did not approach the prey again, and therefore we did not consider them as habituated.

The number of encounters with the moving prey before birds reached the habituation criteria varied from 0 (no behavioral responses during the first encounter) to 10. We did not find any differences in the habituation speed among the deimatic behavior treatments (Kruskal–Wallis: χ^2^ = 0.237, df = 2, *P* = 0.89, Figure S1a) but our low sample size (*n* = 14) meant that this analysis had low statistical power. We also found no evidence that the speed of habituation to the moving prey was associated with the speed of habituation to the robot sound during the control trials (Spearman's correlation: rho = 0.045, *P* = 0.88). For the birds that did not habituate to the movement, the number of encounters with the moving prey before rejection varied from 1 to 5. This did not differ among the treatments (Kruskal–Wallis: χ^2^ = 0.057, df = 2, *P* = 0.97, Figure S1b) but the sample size in the analysis was again low (*n* = 18).

## Discussion

Prey deimatic behaviors often include multiple components that could have interactive effects on predator behavior (Rowe and Halpin 2013; Drinkwater et al. 2022). Using a robot moth baited with cheese and Australian magpies as predators, we found that movement alone, without further defenses such as chemical secretions or warning colors, was enough to significantly reduce predator attacks during the first encounter with the prey. This result provides support for the hypothesis that sudden movement of the prey has protective value against naïve predators, which could allow the evolution of other defenses that further increase the efficacy of deimatic behavior (startle-first hypothesis; Umbers et al. 2017). Whether the moving robotic moth remained small, remained large or increased in size during the encounter, did not have a significant effect on the birds' first responses to the movement. However, an apparent size change might make birds more hesitant to return to the prey after the movement, although these results should be interpreted with caution, as the difference between treatments was not statistically significant. Our results highlight the importance of investigating both initial predator responses and repeated interactions with prey if we are to understand how different components of deimatic behaviors influence prey survival.

Many deimatic behaviors include sudden movements when prey open their wings or lift their body, legs or a tail (eg Brodie 1977; Martins 1989; Umbers and Mappes 2015; Kang et al. 2017a; O’Hanlon et al. 2018). Our finding of this movement alone stopping a predator attack is similar to the study by Holmes et al. (2018) which demonstrated that prey wing movement increased the attack latency of domestic chicks. However, contrary to their experiment that included continuous wing flicking movement (Holmes et al. 2018), in our study the prey performed only one sudden movement, and even this was enough to deter most of the predators during the first encounter with the prey. One potential confounding factor in our study is the mechanical sound of the robot that we could not exclude, and that could potentially have interactive effects with movement. In other studies, sound alone has been demonstrated to influence predator responses, including caterpillar whistles (Dookie et al. 2017) or hissing sounds and ultrasonic clicks of a peacock butterfly (Olofsson et al. 2012). In our study all birds were pretrained and habituated to the robot sound before the movement trials, and we found during this pretraining that most birds did not show strong behavioral responses to the sound. The robot sound is therefore unlikely to influence our results, but future studies where the sound is eliminated are needed to confirm this.

After being presented with the moving robot, magpies often performed typical startle behaviors that have been observed also in other deimatic behavior studies (Schlenoff 1985; Ingalls 1993; Kang et al. 2017b; Holmes et al. 2018; Umbers et al. 2019), including hopping or leaning away from the prey and flapping wings. The cognitive processes underlying these predator responses to deimatic behavior remain poorly understood (Penacchio et al. 2025). There are several potential sensory and cognitive mechanisms that prey deimatic behavior could exploit (Drinkwater et al. 2022; Penacchio et al. 2025). These include a startle reflex which is an immediate response to a sudden intense stimulus and makes an animal interrupt any ongoing activity (Koch 1999), a looming reflex which is an evasive response to avoid contact with rapidly approaching objects (Yamawaki 2011), and a fear response where a sudden stimulus is classified as a potential threat such as a predator (De Bona et al. 2015). In addition, a rapid onset of sensory information in deimatic behaviors could overwhelm a predator's ability to process this information and lead to sensory overload (Hebets and Papaj 2005). Investigating which of these cognitive mechanisms underlie predator responses to deimatic behavior is important for understanding the protective value of the defense; however, disentangling different mechanisms based on behavioral observations is difficult (Drinkwater et al. 2022; Penacchio et al. 2025).

While we found no effect of prey size or size change on predator initial response, we found a possible trend that apparent size change from small (2 wings visible) to large (4 wings visible) increases the latency to return to the prey after the moving display, although this effect was marginally nonsignificant (*P* = 0.051). The apparent size change might also be important for preventing predator habituation, but we did not detect statistically significant differences between the treatments, possibly because of our low sample size. Cognitive mechanisms responsible for predator habituation remain unknown. A sudden increase in size could trigger a looming reflex (Yamawaki 2011; Drinkwater et al. 2022) or make the prey look more intimidating, but these ideas need to be experimentally tested. Future work should also investigate individual variation in predator responses and habituation. We found that some individuals attacked the prey immediately or habituated to the display after just 1 encounter, whereas others needed several encounters or did not habituate at all. Similar individual differences in responses to deimatic behavior have been found in blue jays (Ingalls 1993). These could be explained by several factors, such as birds' age, sex, personality and previous experience, but we were not able to investigate these factors in our study because we used free-ranging wild birds that needed many days to complete the experiment, which limited the number of individuals we could test. Furthermore, different predator species are likely to differ in their responses to deimatic behavior (Drinkwater et al. 2022), and more studies with different predator species across a range of taxa is needed to gain a better understanding of the protective value of deimatic behaviors.

Finally, even though we conducted our experiment in the field with wild predators, 1 limitation of the study is that birds had to hop onto the experimental arena to see the prey. This means that the birds always encountered the prey in the same place, and they could have associated the arena with the moving prey, which could reduce the effect of surprise. This and the frequent encounters with the same prey might have increased the speed of habituation, and it is possible that the birds would have been less likely or slower to habituate to the movement if the prey was presented less frequently and in varying locations to minimize birds' prior expectations. Previous studies on predator habituation with captive predators have similar limitations (Ingalls 1993), and investigating predator habituation in more natural conditions over a longer time scale remains a challenge for future studies.

Although deimatic behaviors are often considered as a textbook example of antipredator defenses, there are still many unresolved questions about their survival value and evolution (Drinkwater et al. 2022). Here, we have shown that sudden movement alone, without typical warning colors, may increase prey survival by preventing or delaying the attack of naïve avian predators. This provides support for the idea that deimatic behaviors can evolve in nonwarningly colored species that avoid predator attacks by rapid movements (startle-first hypothesis, Umbers et al. 2017). We also found that some predators quickly habituated to the moving display, but the likelihood and speed of habituation varied highly among the tested predator individuals, and future studies should aim to identify the mechanisms underlying this variation. Research on predator responses to deimatic behaviors is also still limited to a small number of species with most evidence coming from avian predators (eg Schlenoff 1985; Ingalls 1993; Kang et al. 2017b; Holmes et al. 2018; Umbers et al. 2019; but see Olofsson et al. 2012), and expanding the studies to other predator taxa is crucial for understanding the selection pressures for deimatic behavior in different predator–prey communities.

## Supplementary Material

arag047_Supplementary_Data

## Data Availability

Analyses reported in this article can be reproduced using the data and the code provided by Hämäläinen et al. (2026).
