# Supplementary material for: The protective value of the size and movement components of deimatic behavior
Source: Behav Ecol. 2026 Apr 30;37(4):arag047. doi: 10.1093/beheco/arag047 (PMC13190295; doi:10.1093/beheco/arag047)
Supplement: arag047_Supplementary_Data [file arag047_supplementary_data.zip › Supplementary material.docx]

**SUPPLEMENTARY MATERIAL FOR**

**The protective value of the size and movement components of deimatic behavior**

**Liisa Hämäläinen^1,2*^, Connor Marsland^1^, Thomas E. White^3^, Hannah M. Rowland^4^ & Kate D.L. Umbers^1,5^**

1. School of Science, Western Sydney University, Penrith 2751, NSW, Australia
2. Department of Biological and Environmental Science, University of Jyväskylä, 40014 Jyväskylä, Finland
3. School of Life and Environmental Sciences, The University of Sydney, Sydney, 2006, NSW, Australia
4. Department of Evolution, Ecology and Behaviour, Institute of Infection, Veterinary and Ecological Sciences, University of Liverpool, Liverpool, UK
5. Hawkesbury Institute for the Environment, Western Sydney University, Penrith 2751, NSW, Australia

***Correspondence:**

Liisa Hämäläinen

Department of Biological and Environmental Science, University of Jyväskylä

Email: liisa.l.hamalainen@jyu.fi

**Supplementary figures: predator habituation and prey rejection**

**
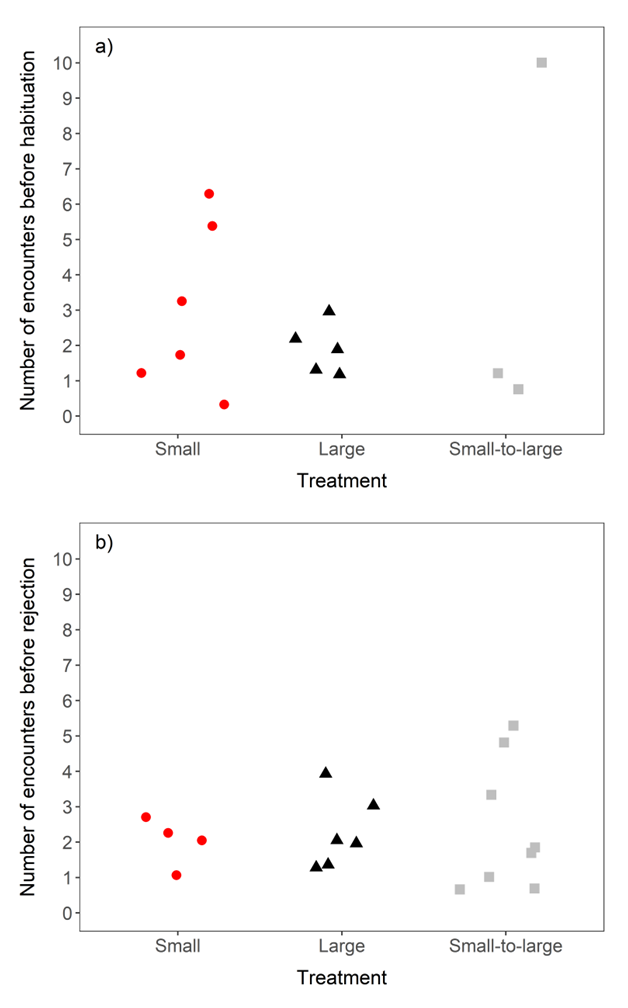
**

Figure S1. The number of encounters with the moving prey before birds either a) habituated to the movement, i.e. showed no behavioral responses in two consecutive trials (small: n = 6, large: n = 5, small-to-large: n = 3), or b) did not approach the prey again in 4 x 15min experimental trials (small: n = 4, large: n = 6, small-to-large: n = 8).
